# The Principles and Practice of Surgery

**Published:** 1886-08

**Authors:** 


THE PRINCIPLES AND PRACTICE OF SURGERY. By Frank Hastings Hamilton, A. M., M. D., LL. D. Third edition, revised and corrected. New York: Wm. Wood & Co. 1886.

The appearance of a new edition of this distinguished surgeon's work has been looked for with eagerness by all specially interested in surgery. The second edition appeared in 1873, and in this long interval of thirteen years, surgery has advanced with such rapid strides that we felt curious to know what judgment the conservative mind of Dr. Hamilton would pass on recent surgical procedures. A perusal of his book will convince one that he has not departed from his well-known conservatism. His acknowledged eminence as an authority in surgery gives us great respect for his views, even where he opposes the large majority of modern surgeons.

In the opening chapters there is very little deviation from generally accepted views. These chapters treat of Inflammation, Abscess, Ulceration and Gangrene. In the chapter devoted to ulceration there is a succinct and instructive article on the "Healing of Ulcers by Transplantation."

When we reach chapter V. our interest becomes at once fixed. The treatment of wounds is discussed in this chapter, and we find that the distinguished author still refuses to give his adherence to antiseptic surgery. Nay, more: In a supplementary chapter at the end of the book, on "The Art of Primary Union, or Union by Adhesion in large incised Wounds," he ably defends the plan of treatment adopted by Alanson, of Liverpool, in 1779, and makes a vigorous attack on Lister's theories. He ascribes Lister's success entirely to the fact that he carried out, in detail, the principles enunciated by Alanson nearly an hundred years before, and regards all his antiseptic precautions as "serving no other purpose than the walking, talking and gestures of the prestidigitator. They abstract the attention and conceal the adroit manipulation by which the trick is actually performed." He quotes Ashhurst, Keith, Lawson Tait and others in support of his position. The great success of Tait and Keith in the field of abdominal surgery is one of his strong arguments. He also considers it "unsound practice to search for a wound in the intestines with the purpose of closing its edges with sutures," and says that "antisepsis has in no degree abated the dangers and difficulties" of these operations. In a paper published in the New York *Medical Record*, January 30, 1886, Dr. Simon Baruch, of New York, ably answers Dr. Hamilton, and shows that the statistics of antiseptic surgery have never been equaled. Space does not allow quotations from Dr. Baruch's paper, but it will well repay perusal. Dr. Hamilton and Dr. Ashhurst, on this side of the Atlantic, and Lawson Tait and Mr. Keith on the other side, are the most distinguished opponents of Listerism. The vast majority of English, American and German surgeons advocate and practice it. The subject of wounds is concluded by an interesting and exhaustive article on Gunshot Wounds. Fractures and dislocations are handled in a most masterly and practical manner. It is gratifying to see that, in writing of the treatment of special fractures, the author has not loaded his text with manifold illustrations of splints. He gives, under each fracture, one or more methods of procedure that he can recommend from personal experience, and does not confuse the student by picturing all the splints that have ever been devised. Part I. closes with a consideration of tumors, which are classified from their clinical rather than from their anatomical features. This is the old classification so familiar in

English and American text-books, and it is probably the best one for a strictly surgical work.

Part II. is devoted to Regional Surgery, and the best chapter in this part is the one on Vesical Calculi.

In conclusion, the book is heartily recommended to the student and practitioner; for the former it is a most excellent and complete text-book; for the latter a safe and sure guide in every-day practice. If at times the author is dogmatic in his teachings, it is because he is a man with a vigorous mind and thinks for himself. His conclusions are logical deductions from his experience, and the whole work bears the impress of his originality.

